# The 100-plus Study of cognitively healthy centenarians: rationale, design and cohort description

**DOI:** 10.1007/s10654-018-0451-3

**Published:** 2018-10-25

**Authors:** Henne Holstege, Nina Beker, Tjitske Dijkstra, Karlijn Pieterse, Elizabeth Wemmenhove, Kimja Schouten, Linette Thiessens, Debbie Horsten, Sterre Rechtuijt, Sietske Sikkes, Frans W. A. van Poppel, Hanne Meijers-Heijboer, Marc Hulsman, Philip Scheltens

**Affiliations:** 1 Alzheimer Center Amsterdam, Department of Neurology, Amsterdam Neuroscience, Vrije Universiteit Amsterdam, Amsterdam UMC, de Boelelaan 1118, 1081 HZ Amsterdam, The Netherlands; 2 Department of Clinical Genetics, Amsterdam Neuroscience, Vrije Universiteit Amsterdam, Amsterdam UMC, de Boelelaan 1118, 1081 HZ Amsterdam, The Netherlands; 3 Netherlands Interdisciplinary Demographic Institute (NIDI/KNAW), Lange Houtstraat 19, 2511 CV The Hague, The Netherlands

**Keywords:** 100-plus Study, Prospective cohort study, Centenarians, Cognitive health longevity

## Abstract

Although the incidence of dementia increases exponentially with age, some individuals reach more than 100 years with fully retained cognitive abilities. To identify the characteristics associated with the escape or delay of cognitive decline, we initiated the 100-plus Study (www.100plus.nl). The 100-plus Study is an on-going prospective cohort study of Dutch centenarians who self-reported to be cognitively healthy, their first-degree family members and their respective partners. We collect demographics, life history, medical history, genealogy, neuropsychological data and blood samples. Centenarians are followed annually until death. PET–MRI scans and feces donation are optional. Almost 30% of the centenarians agreed to post-mortem brain donation. To date (September 2018), 332 centenarians were included in the study. We analyzed demographic statistics of the first 300 centenarians (25% males) included in the cohort. Centenarians came from higher socio-economic classes and had higher levels of education compared to their birth cohort; alcohol consumption of centenarians was similar, and most males smoked during their lifetime. At baseline, the centenarians had a median MMSE score of 25 points (IQR 22.0–27.5); most centenarians lived independently, retained hearing and vision abilities and were independently mobile. Mortality was associated with cognitive functioning: centenarians with a baseline MMSE score ≥ 26 points had a mortality percentage of 17% per annual year  in the second year after baseline, while centenarians with a baseline MMSE score < 26 points had a mortality of  42% per annual year (*p* = 0.003). The cohort was 2.1-fold enriched with the neuroprotective *APOE*-*ε2* allele relative to 60–80 year-old population controls (*p* = 4.8 × 10^−7^), *APOE*-*ε3* was unchanged and the *APOE*-*ε4* allele was 2.3-fold depleted (*p* = 6.3 × 10^−7^). Comprehensive characterization of the 100-plus cohort of cognitively healthy centenarians might reveal protective factors that explain the physiology of long-term preserved cognitive health.

**Electronic supplementary material:**

The online version of this article (10.1007/s10654-018-0451-3) contains supplementary material, which is available to authorized users.

## Background

Although increasing age is the strongest risk indicator for cognitive decline and dementia, it is not an inevitable consequence of aging. The incidence of overall dementia starts to increase exponentially from approximately 60 years and at age 100 years the annual dementia incidence reaches 40% per year [1, 2]. However, the mere existence of cognitively healthy individuals older than 110 years [3–6] leads to the intriguing suggestion that the incidence of dementia decelerates somewhere after 100 years (see Box). Factors that allow for the preservation of cognitive health may thus be enriched for in super-agers, individuals who reach extreme ages with full cognitive functions [7]. The combination of extreme old age with maintained cognitive health is often observed in families [8–13], suggesting that beneficial factors involved in the long-term maintenance of both cognitive and overall health are heritable, and likely genetic [7, 14–16]. Indeed, results from the New England Centenarian Study indicated that siblings from centenarians are ~ 8–12 times more likely to reach 100 years compared to individuals with no centenarian siblings [17].

This raises several questions: what are the unique molecular mechanisms that cause resilience against age related decline? Which hereditable factors are involved, and what is the role of the immune system? The answers to these questions are likely to provide novel insights in the effects of aging on the brain and they will be informative for the design of novel strategies that intervene in processes that lead to neurodegenerative diseases [18]. Answers to these questions might be found in the context of prospective follow-up studies, however, this is complicated by the fact that only ~ 0.6% of the population born in the early 1900s reaches 100 years (see Box). Therefore, we set out to identify protective factors in a cohort of centenarians who self reported to be cognitively healthy. For this, we initiated the 100-plus Study in 2013 at the Alzheimer Center Amsterdam in the Netherlands (www.100plus.nl). To date, the cohort includes 332 centenarians.

Children of centenarians also profit from the advantage they inherited from their centenarian parent: they live longer, and they have almost a 90% lower risk of developing myocardial infarction, stroke and diabetes compared to age-matched peers whose parents have average life spans [15, 19, 20]. Together, this suggests that first-degree family-members of centenarians are also enriched for protective (genetic) factors and that efforts to identify protective factors should include targeting the families of centenarians [21]. The value of using by-proxy phenotypes for genetic studies was recently demonstrated for 12 diseases [22], and recently for Alzheimer’s Disease [23, 24]. Centenarian children represent the by-proxy phenotype of extreme longevity which allows the opportunity to compare the composition of biomaterials or other features relative to age-matched controls [25]. For this reason, we extended the 100-plus Study with a second phase in 2017, in which we also include first-degree family-members of centenarians and their partners.

The 100-plus Study has a main focus on the biomolecular aspect of preserved cognitive health. It is beneficial that cohort inclusion is on-going, as this allows us to take optimal advantage of the recent developments in high-throughput biomolecular techniques. For example, genetic variants of interest can be functionally tested in our collection of fresh blood samples and brain tissues from carriers.

Here we present the rationale for the 100-plus Study (see Box), we describe the study design and procedures, and we introduce the 100-plus Study cohort based on the clinical presentation of the centenarians at baseline, and the demographic characteristics of the centenarians relative to their birth cohort.

## Methods/study design

Please find in the electronic supplementary material (ESM.pdf) a complete compendium of participant inclusion procedures and current data collection procedures of the 100-plus Study.

### Inclusion and exclusion criteria

The 100-plus Study includes (1) Dutch-speaking centenarians who can (2) provide official evidence for being aged 100 years or older, (3) self-report to be cognitively healthy, which is confirmed by an informant (i.e. a child or close relation), (4) consent to donation of a blood sample and (5) consent to (at least) two home-visits from a researcher, which includes an interview and neuropsychological testing. In the second phase of the 100-plus Study (from September 2017 onwards) we include (1) siblings or children from centenarians who participate in the 100-plus Study, or partners thereof who (2) agree to donate a blood sample, (3) agree to fill in a family history, lifestyle history and disease history questionnaire. Study exclusion criteria are limited to subjects who are legally incapable.

### Recruitment and research visits

#### Recruitment

We regularly perform an online search for local newspaper articles that mention a centenarian. These reports commonly include the name of the centenarian, and sometimes a description of their well-being and living situation. We retrieve an address online and we approach a prospective study participant by letter. When they express their interest in study participation and inclusion criteria are met, we schedule two baseline visits. (See ESM.pdf for detailed recruitment procedures).

#### Baseline visit

**Fig. 1:**
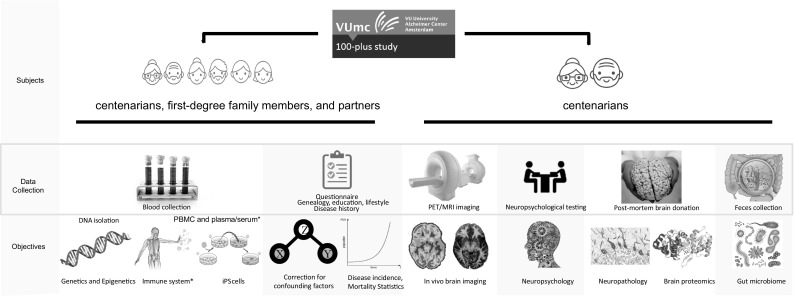
Overview of the 100-plus Study, Phase 2: During home visits we inquire about life-history of the centenarians, their family history, medical history, and current health. We assess their performance on neuropsychological tests, measure blood pressure and grip strength and we collect a blood sample, for blood testing and genetic analyses. Optional parts of the study are: a visit to the outpatient clinic for PET–MRI and/or PET–CT imaging, feces donation to investigate the gut microbiome, and the generation of iPS cells from peripheral blood. Furthermore, all participants are informed about the option of post-mortem brain donation in collaboration with the Netherlands Brain Bank [37]. This is optional and not required for study participation. We evaluate changes in general well-being and in neuropsychological test performance during (half-)yearly follow-up visits. Next to the centenarians, we also include their first-degree family members and their partners. *Collected in Phase-2 of the 100-plus Study, started in September 2017

A researcher, trained to perform standardized visit procedures, will visit the centenarian. The baseline visit (T0) consists of two visits. The first baseline visit takes approximately 2 to 3 h, and comprises obtaining informed consent for study inclusion, a life-history interview, an assessment of genealogy, and an assessment of current health and medical history (Table 1). The second baseline visit, approximately 1 week after the first, takes approximately 1.5 h: during this visit we subject the centenarian to a battery of neuropsychological tests and we measure grip strength and blood pressure (Table 2). During the first baseline visit we inform participants of optional parts of the 100-plus Study: feces collection, PET–MRI or PET–CT imaging and post-mortem brain donation (Fig. 1). Once a centenarian volunteers to participate in these parts of the 100-plus Study, we obtain informed consent for these study parts separately.

**Table 1:** Overview of 100-plus Study data-collection

Study participants	Actions
**Centenarians**	**First baseline visit: interview**
Phase 1 and Phase 2	Formalities for study inclusion: ICF; Proof of age
	Childhood living environment; Education; Marriage/Partners; Number of children, Religion, Occupation; Occupation of parents and partner
	Genealogy of first degree family members and partners; Disease history in family
	Lifestyle Questionnaire: Smoking habits; Drinking habits; Lifetime cognitive activity scale; situation during WWII
	Disease history (self-report): weight/length; incontinence; medication intake, dental condition (stopped); hospital visits/anesthesia
	Researcher subjective estimate of sight, hearing, mobility, cognitive status;
	Centenarian presentation: current housing situation, total hours of care; ADL (Barthel index); sleep quality (PSQI); Geriatric Depression Scale (GDS); cognitive well-being judged by informant (IQ-CODE)
	Collection of biomaterials and biomarkers: blood sample^ab^
	**Second baseline visit**
	Neuropsychological test battery: Table 2
	Measurement of grip strength^b^ and blood pressure^b^
	**Follow up**
	MMSE at last visit > 20: yearly visit: update of general well-being, disease history, and missed items at baseline interview; Researcher subjective estimate of sight, hearing, mobility, cognitive status;
	Neuropsychological testing battery (Table 2) Barthel index; GDS; IQ-CODE, grip strength measurement^b^; blood pressure measurement^b^
	MMSE at last visit ≤ 20; phone interview: update of general well-being, disease history, and missed items at baseline interview; IQ-CODE (by mail), ADL (Barthel index)
	For brain donors: half yearly follow-up: TICs-M (by telephone); IQ-CODE (by mail)
	**GP**
	At baseline inclusion: request for summary of medical events
	Post mortem: request medical events leading to death
	**Optional in Phase-2**
	MRI-PET or PET-CT scan
	Feces donation
	iPS cell generation
	Post mortem brain donation
Centenarian children and partners Phase-2	**Baseline visit**
	Formalities for study inclusion: ICF
	Collection of blood sample^b^
	Mail: Questionnaire on lifestyle, general well-being, education and occupation, disease history and genealogy
	**Follow up**
	No follow-up
	**GP**
	For specific cases: request for summary of medical events
Centenarian-siblings and partners, centenarian-partners Phase-2	**Baseline visit**
	Update lifestyle questionnaire, current health, disease history and general well-being Blood sample, MMSE, Barthel index; IQ-CODE; grip strength^b^ and blood pressure measurement,^b^ estimation of sight, hearing, and mobility;
	Researcher subjective estimate of sight, hearing, mobility, cognitive status;
	Mail: questionnaire on lifestyle, general well-being, education and occupation, disease history, and genealogy
	**Follow up**
	Yearly: TICs-M (by telephone); IQ-CODE (by mail)
	**GP**
	For specific cases: request for summary of medical events

^a^Blood sample collection may occur at a different occasion, close to first baseline visit; Phase-2 of the 100-plus Study started in September 2017

^b^Blood sample biomarkers determined in the blood sample, assessment of blood pressure and measurement of grip strength are described in detail in ESM.pdf. TICS-M: Telephone Interview Cognitive Status—Modified (see Table 2); IQ-COde Informant Questionnaire on Cognitive Decline in the elderly short form (see Table 2)

**Table 2:** Neuropsychological tests and questionnaires

Domain or goal	Assessment/questionnaires	Duration (min)
**Cognitive functioning**		
Overall cognitive functioning	Researcher subjective impression of cognitive health (see “Methods”)	0
	Mini–Mental State Examination [26, 86]	5
	National Adult Reading Test^a^ [88–89]	3
	Telephone Interview Cognitive Status—Modified (TICS-M)^d^ [90]	
Memory	CERAD 10-word list—immediate and delayed recall [91]	15
	Visual Association Test—Memory [92]	5
	Rivermead Behavioral Memory Test (RBMT)^b^ immediate and delayed recall [93, 94]	6
Attention	Digit Span—forwards [96–97]	3
	Trail Making Test A [98, 99]	NA
Executive functions	Digit Span—backwards [96–97]	3
	Letter Fluency—DAT [101–104]	2
	BADS—subtest Key Search [105, 106]	3
	BADS—subtest Rule Shift Cards [105, 106]	3
	Trail Making Test B [98, 99]	10
	Amsterdam Dementia Screening Test—Meander figure [107]	2
Language	Category Fluency—Animals [100, 101, 108]	2
	Visual Association Test—Naming [92]	1
Visuo-spatial functioning/construction	CAMDEX-R/N CAMCOG—figure copying [109, 110]	3
	Clock Drawing Test [111, 112]	2
	Visual Object and Space Perception (VOSP) Battery^b^—subtest Number Location [113]	3
**Depression, ADL, sleep, lifestyle, geriatric impairments**		
Depressive symptoms	Geriatric Depression Scale-15 (GDS) [29]	4
(Instrumental) Activities of daily living	Informant Questionnaire on Cognitive Decline in the elderly short form (IQ-CODE) [30, 31]	3
	Barthel Index [27, 115–116]	3
Lifetime cognitively stimulating experience	Lifetime Cognitive Activity Scale^a^ [34, 35]	5
Sleep quality	Pittsburgh Sleep Quality Index^a^ (PSQI) [28]	5
Geriatric impairments	Researcher subjective impression of sight, hearing, mobility (“Methods”)	0

^a^Only administered at baseline, ^b^ In 100-plus Study-phase 1 only, ^c^ Included with the confirmation letter of study-inclusion, collected during the first baseline visit, ^d^ Only administered during half yearly-follow up of brain donors and yearly follow-up of siblings

#### Follow-up visits

**Fig. 2:**
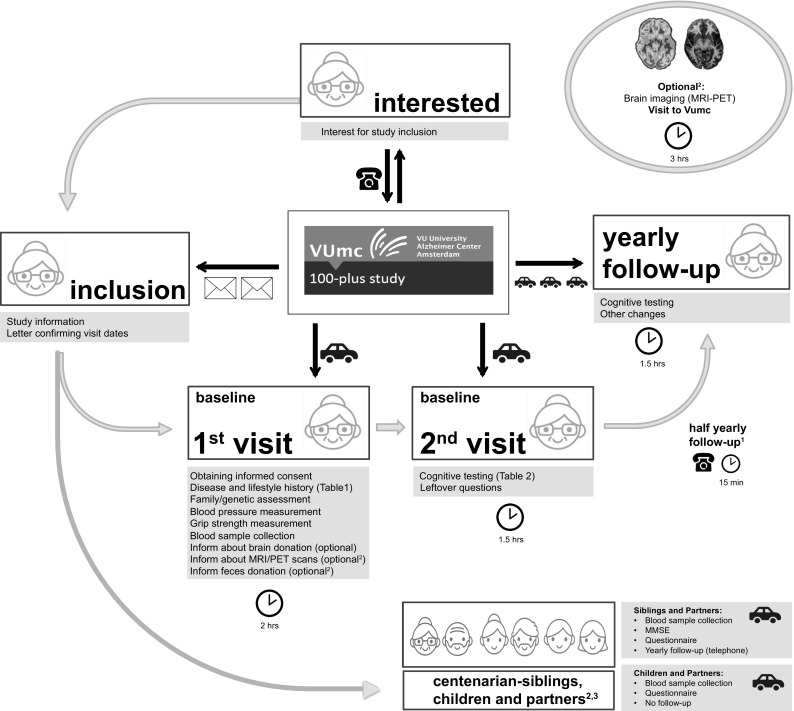
Diagram of visit procedures of 100-plus Study: ^1^Half yearly follow-up by telephone is performed for centenarians who agreed to brain donation. ^2^Collected in phase-2 of the 100-plus Study, started in September 2017. ^3^Data from centenarian-children and children in-laws will be obtained during the visit with the centenarian

During yearly follow-up visits (T1, T2, …), which take approximately 2 h, we inform about possible changes in cognitive functioning that took place in the last year, we update the interview questionnaire and re-administer the complete cognitive test battery and physical measurements (Tables 1, 2). Follow-up is continued until the participant is no longer willing/able to participate. When the MMSE score declines ≤ 20 there is evidence of clear cognitive impairment [26], and subjecting a centenarian to a neuropsychological testing battery becomes more complicated and follow-up visits by a researcher may no longer be constructive. When the MMSE at last visit drops below 20 (imputed MMSE score), we follow-up by informant questionnaire. To ensure up-to-date cognitive health measurements of brain donors, we administer telephone an informant questionnaires 6 months after the annual visit (T0.5, T1.5, …). For a diagram of procedures see Fig. 2. We ask informants to inform us when a participant dies and about the events that preceded death.

**Fig. 3:**
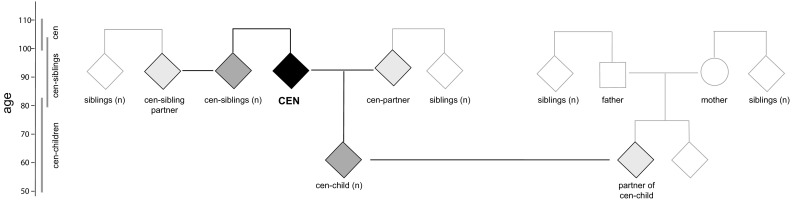
Data collection from centenarians and their family-members: In Phase-2 of the 100-plus Study (since September 2017), we obtain blood-samples from centenarians (black), and when willing, their siblings, their children (dark grey) and their respective partners (light grey). We will inquire about longevity and incidence of dementia in relatives from the same generation as the centenarian (white). Square: male, circle: female, diamond: both genders are possible

### Data collection

#### Centenarian presentation

During each visit, the researcher subjectively estimates the visual, hearing and mobility function as “good”, “moderate”, “poor” or “very poor”, according to the determinants listed in Table 3. We collect the following variables regarding centenarian presentation: the level of independence during activities of daily living (ADL) using the Barthel Index [27], an estimation of the total hours of care/assistance needed *per week*; category housing situation, (independent-dependent); grip strength, systolic and diastolic blood pressure; heartbeat; and napping habits and sleep quality (Pittsburg Sleep Quality Index questionnaire [28]). We assess whether the centenarian suffers from symptoms of depression [29] by administering the 15-item geriatric depression scale (GDS-15). We ask about recent weight loss, current weight and length and whether the centenarian has active infections.

**Table 3:** Categorization of vision, hearing and mobility ability

	Vision	Hearing	Mobility
Good	Able to read newspapers and watch television	Able to have and follow a conversation in a group of people	Able to walk independently (with or without help of a walking stick or walker)
Moderate	Able to read large texts with large letters and watch television	Able to have a conversation with one person/questions do not have to be repeated	Able to walk with help of another person
Poor	Not able to watch television/vision problems cause some difficulties in ADL	Limited ability to have a conversation with one person/questions need to be repeated multiple times	Able to move independently in a wheelchair
Very poor	Limited or complete loss of vision which causes severe difficulties in ADL	Not able to have a conversation with one person; this does not improve when speaking loud and clearly	Not able to move independently in a wheelchair

Vision and hearing abilities were estimated while participants used all available devices to support their vision and/or hearing

#### Medical history

From the General Practitioner (GP) of the centenarian, we request a summary report of the diagnosed conditions and prescribed medications. These conditions are categorized by a GP dedicated to the 100-plus Study (Table 4). After a centenarian dies, we request a second synopsis from the GP, describing the medical proceedings until death. In a self-report medical history questionnaire, we inquire about blood pressure, heart disease; stroke (CVA) or TIA; tumors, head injuries, incontinence, dental condition, mental health problems, hospital visits, surgeries/anesthesia. We estimate BMI at midlife by recording self-reported weight and length at middle-age (~ 50 years). For centenarian-females we inquire about age at menarche, onset of menopause, number of pregnancies and/or miscarriages.

**Table 4:** Categories of conditions analyzed in the GP medical files of 209 centenarians

Condition-category	Conditions
Fraction of centenarians with at least one mention of this condition in their GP report (%)	Fraction of centenarians with a at least one mention of these conditions in their GP report (%)
Cardiovascular disease (83.7%) Cardiovascular disease without hypertension (66.5%)	Hypertension (48.8%); congestive heart failure (29.7%); cardiac dysrhythmia (23%); CVA/TIA (18.7%); angina pectoris (15.3%); myocardial infarction (8.1%); valvular heart disease (8.1%); thrombosis (6.2%); pacemaker (5.7%); aortic stenosis (2.9%); amputation leg (1.4%); coronary bypass (1%); hypercholesterolemia (1%); arterial disease (0.5%); arteritis temporalis (0.5%); atherosclerosis (0.5%); cerebrovascular insufficiency (0.5%); coronary sclerosis (0.5%); intermittent claudication (0.5%); orthostatic hypotension (0.5%); pericarditis (0.5%)
Musculoskeletal (63.2%)	Arthrosis (35.4%); fractures (34.4%); osteoporosis (14.8%); joint(s) replacement (11.5%); osteoarthritis (3.3%); hernia (1%)
Vision (41.6%)	Cataract (30.1%); macular (7.7%); glaucoma (3.8%); vision impairment (2.4%)
Hearing (30.6%)	Hearing impairment (30.6%); cholesteatoma (0.5%); sudden deafness (0.5%)
Cancer (27.8%)	Skin cancer (17.2%); breast cancer (4.3%); colon cancer (4.3%); prostate cancer (1.9%); uterus cancer (1.4%); bladder cancer (0.5%); choleasteatome (0.5%); palate cancer (0.5%); stomach cancer (0.5%); thyroid cancer (0.5%); vocal chord cancer (0.5%)
Autoimmunology (22%)	Diabetes (7.7%); rheumatoid arthritis (4.8%); hyperthyroidism (3.8%); hypothyroidism (3.3%); skin cancer (1.4%); asthma (1%); hypopituitarism (0.5%); thyroid enlargement (0.5%); thyroid removal (0.5%)
Urology (21.5%)	UTI (7.2%); incontinence (5.7%); prostate hypertrophy (4.8%); hysterectomy (1.9%); uterine prolapse (1.9%); catheter (1%); prostate resection hypertrophy (1%); ovarian cysts (0.5%);
Neurology/psychiatry (15.8%)	Balance (3.3%); cognitive decline (2.9%); depression (2.4%); psychiatry (2.4%); epilepsy (1.9%); delirium (1.4%); insomnia (1%); Parkinson’s (1%); dizziness (0.5%); migraine (0.5%); tremor (0.5%); WM atrophy (0.5%)
Gastrointestinal (15.3%)	Kidney failure (6.7%); gastric ulcer (1.9%); cholecystectomy (1.4%); diverticulosis (1.4%); gall stones (1.4%); kidney stones (1%); reflux esophagitis (1%); appendectomy (0.5%); intestinal polyps (0.5%); pancreatitis (0.5%); rectal prolapse (0.5%); sigmoid resection (0.5%)
Lung disease (10.5%)	Pneumonia (6.2%); COPD (2.4%); TBC (1.9%); Emphysema (0.5%); ulcer (0.5%)
Other	Erysipelas (1.4%); anemia (1%); herpes zoster (1%); other (1%); restless legs (1%); eye infection (0.5%); itching (0.5%); pes equinus (0.5%); vitamin B deficiency (0.5%); vitiligo (0.5%)

Left column: when multiple conditions that belong to one condition-category are mentioned more than once in the GP report of a centenarian, they are counted as one. Right column: all conditions are counted separately, even though they belong to one condition-category. In aggregate, the percentages in the right column will exceed the percentage in the left column

#### Cognitive profiling

We objectively evaluate cognitive functioning using a comprehensive neuropsychological test battery that addresses memory, attention and/or concentration, pre-morbid intelligence, language, executive and visuo-spatial functions (Table 2). To assess overall cognitive functioning we administer the Mini–Mental State Examination (MMSE) [26]. Geriatric sensory impairments such as bad eyesight or bad hearing complicated performance, which led to missing items. MMSE scores with different missing items cannot be directly compared, because the total obtainable score is different per centenarian. Therefore, we adjust scores using multiple imputation (see ‘MMSE imputation’ in ESM.pdf). In addition, at every visit the researcher subjectively estimated cognitive functioning of the centenarian (for procedures see ESM.pdf). During each research visit we ask an informant to fill in the Dutch version of the abbreviated form of the Informant Questionnaire on Cognitive Decline (IQ-CODE) to indicate whether the centenarians experienced cognitive decline in the past 10 years (or, in case of follow-up visits, during the past year) [30, 31].

#### Lifetime/demographic characteristics

To investigate the family genealogy and disease occurrence, we draw a pedigree including children, siblings, parents and grandparents, their (maiden) names, gender, birth years, age at death and cause of death, occurrence of dementia/cognitive decline (Fig. 3). To determine socio-economic background (SEB) and socioeconomic status (SES) we inquire about the main occupation of the father and mother of the centenarian, the main occupation of the centenarian him/herself at adulthood and the main occupation of their partner(s). We inquire about the education level and the number of years education was followed. Education levels were classified according to (I) ISCED 1997 [32] and according to the classification system used in the Dutch 1971 census [33].

#### Lifetime habits

We address smoking habits and alcohol consumption (see ESM.pdf). We administer the Cognitive Activity Questionnaire (CAQ) [34, 35] to investigate cognitive stimulating experience during adult life (from childhood to 50 years) and current cognitively stimulating experience.

#### Data-collection of first degree living centenarian-relatives and partners

For centenarian siblings and their partners, we administer the MMSE at the study inclusion visit and we record the genealogy at the level of the centenarian-generation (Fig. 3). We will yearly monitor changes in physical well-being and in cognitive health using TICS-M and IQ-Code-N. We ask centenarian-children and partners to fill in an abbreviated version of the centenarian questionnaire; we record the genealogy of the centenarian-generation, no cognitive testing will be administered; we will not follow-up centenarian-children and their partners (Table 1).

### Biomaterials

#### Biomaterial collection

We collect a blood sample from centenarians and their family members for DNA isolation, peripheral blood mononuclear cells (PBMCs), plasma, serum, and when consent is given for generation of induced pluripotent stem cells (iPSCs) (Fig. 3). DNA samples are currently used for APOE genotyping, GWAS, whole exome sequencing (WES) and Sanger sequencing. Furthermore, all centenarians are informed about the option for feces donation for gut microbiome analysis, PET–MRI or PET–CT brain scans for in vivo detection of amyloid beta presence and structural brain imaging. We also inform about the option of post-mortem brain donation. Brain autopsies are performed in collaboration with the Netherlands Brain Bank [36, 37]. For numbers of collected biomaterials thus far, please see additional data (ESM.pdf).

#### Data storage

OpenClinica open source software (version 3.1 onwards) is used for data management [38]. Biomaterials are stored in the biobank of the Amsterdam UMC.

## Cohort description

### Included centenarians

**Fig. 4:**
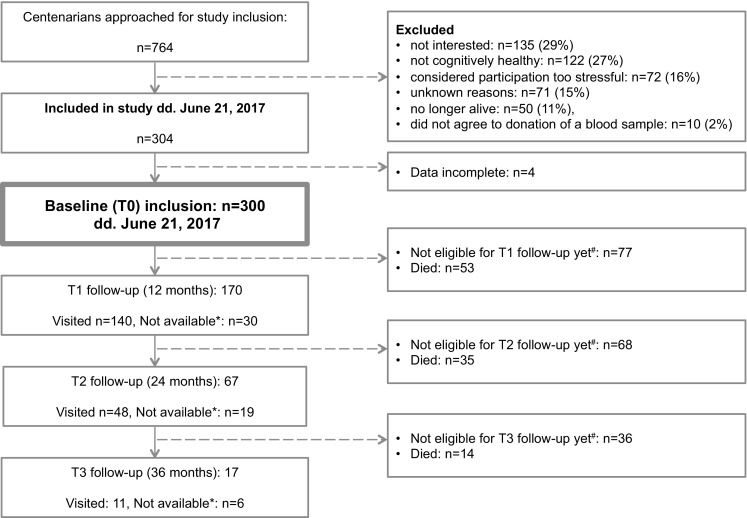
Flowchart of study inclusion: *Not available: centenarians were on vacation, not interested or too frail for a follow-up visit. When possible, follow-up was performed by telephone and/or informant questionnaires. In several cases, centenarians were available for follow-up one year later, such that this ‘unavailable’ group was formally kept in the study until death. ^#^Not eligible: centenarians were not yet included in the study long enough to be eligible for the next follow-up visit

Between January 1st 2013 and September 1st 2018, 332 centenarians were included in the study of whom almost 30% (n = 92) agreed to post mortem brain donation. Thus far, 58 centenarians have come to autopsy. On June 21st 2017, 764 centenarians were approached for study-participation of which 300 (40%) met study-inclusion criteria and were included in the study (Fig. 4). Here, we describe the cohort using the collected data from these first 300 centenarians. For all cohort descriptives see Table 5.

**Table 5:** Descriptive statistics of 100-plus Study cohort

Cohort statistics		
**100-plus cohort, June 2017 (N available, %)**	300	
**Age at inclusion** (mean, SD)	101.3 ± 1.7	
**Birth years** (median, IQR)	1914 (1913–1915)	
**Brain donors** (n, %)	81 (27%)	
**Follow-up visits**		
T0 baseline visits	300	
T1 possible visits (visited, died, missed)	223 (140, 53, 30)	
T2 possible visits (visited, died, missed)	155 (48, 88, 19)	
T3 possible visits (visited, died, missed)	119 (11, 102, 6)	

Mortality		
**Whole cohort**	T0–T1	T1–T2
Mortality rate (95% CI)	0.24 (0.17–0.32)	0.32 (0.20–0.49)
Mortality percentage (95% CI)	21% (16–27%)	28% (18–39%)
**MMSE < 26 at baseline (95% CI)**		
Mortality rate (95% CI)	0.29 (0.19–0.43)	0.54 (0.29–0.90)
Mortality percentage (95% CI)	25% (17–35%)	42% (25–59%)
**MMSE ≥ 26 at baseline (95% CI)**		
Mortality rate (95% CI)	0.19 (0.11–0.29)	0.19 (0.08–0.37)
Mortality percentage (95% CI)	17% (10–25%)	17% (8–31%)

Cognitive functioning at baseline		
**Mini Mental State Examination (MMSE)**		
100-plus cohort (median MMSE, IQR)	25 (22.0–27.5)	
MMSE > 22^a^ (fraction of cohort, %)	72.4%	
MMSE ≥ 26 (fraction of cohort, %)	47.2%	
**Estimated by trained researcher (n = 287)**		
Cognitively healthy (fraction of cohort, %; median MMSE (IQR)	83%; 26 (23.5–28.0)	
Doubt	14%; 19 (16.4–22.0)	
Cognitively impaired	2.8%; 16.4 (12.8–17)	

Baseline presentation		
**Geriatric impairments**		
Mobile: without aids	80.2%	
Hearing: moderate-Good	86.8%	
Vision: moderate-Good	77.1%	
Maintained continence	56.3%	
Number of comorbidities (avg ± SD)	3.7 ± 1.5	
Geriatric depression scale: ≤ 5 (no depression)	91.5%	
**Living independence**		
Community dwelling/private residence with care available	51.9%	
Private quarters in residential care center	42.0%	
**Independence in Activities of Daily Living (Barthel Index)**		
Needs minimal assistance (15–19)	45.1%	
Fully independent (20)	32.4%	

Lifestyle characteristics		
**Smoking: regularly/often**		
Males	67%	
Females	15%	
**Alcohol consumption: regularly/often**		
Males	54%	
Females	31%	

Demographic characteristics		
**Education > basic (primary school)**	Centenarians versus population^b, c^	
Males	79 versus 45%	
Females	66 versus 31%	
**Socioeconomic status**		
SEB: Social class father: ≥ lower-middle class	31.2 versus 17.9%	
SES: Social class-centenarian or –partner: ≥ lower-middle class	55.5 versus 29.4%	
**Number of children parented: (mean ± SD)**	3.9 ± 2.2 versus 3.5 ± 2.5	

APOE genotypes		
**APOE genotypes**	Genotype frequency (%); centenarians versus population^d^	Odds ratio (95% CI); *p* value^e^
*ε2/ε2*	0.9 versus 0.7%	1.30 (0.3–5.7); *p* = 9.6 × 10^−1^
*ε2/ε3*	24.9 versus 11.7%	2.49 (1.8–3.5); *p* = 3.4 × 10^−7^
*ε2/ε4*	4.8 versus 3.0%	1.63 (0.8–3.1); *p* = 2.1 × 10^−1^
*ε3/ε3*	60.3 versus 60.5%	0.99 (0.8–3.1); *p* = 8.9 × 10^−1^
*ε3/ε4*	8.7 versus 21.3%	0.35 (0.2–0.6); *p* = 5.7 × 10^−7^
*ε4/ε4*	0.4 versus 2.9%	0.15 (0.0–1.1); *p* = 3.2 × 10^−3^
**APOE alleles**		
*ε2*	17 versus 10.7%	2.1 (1.6–2.8); *p* = 4.8 × 10^−7^
*ε3*	86.1 versus 87.1%	1.0 (0.8–1.3); *p* = 1.0
*ε4*	3.2 versus 7.5%	0.44 (0.31–0.63); *p* = 6.3 × 10^−7^

^a^An MMSE > 22 is the suggested cutoff score for cognitive health in elderly aged 97 years and above [117], ^b^ Centenarian education levels were compared with 54–61 years olds reported in the Dutch population in the 1971 census [39], ^c^ Socio-economic background was compared with 2815 individuals born between 1910 and 1915 from the Historical Sample of the Netherlands (HSN) [40], ^d^ APOE genotypes were compared with 2233 ~ 50–80-year olds from the Longitudinal Aging Study Amsterdam (LASA) [45], ^e^ *p* values were calculated using a two-sided Fisher’s Exact test

The mean age at inclusion of centenarians was 101.3 ± 1.7 years (ESM.pdf Fig S1A). The majority of centenarians were born between 1910 and 1917 (ESM.pdf Fig S1B). Of the 300 centenarians in the cohort, 284 were born in a Dutch municipality, 6 were born in the Dutch East Indies, (a Dutch colony at the time), and 10 centenarians were born in other European countries. Centenarian birth-municipalities indicated that the catchment area is spread across the 11 provinces of the Netherlands in the early 1900's (ESM.pdf Fig S2).

### Presentation at baseline

Subjective researcher estimates of geriatric sensory impairments indicated that 87% of the centenarians had moderate-good hearing abilities (ESM.pdf Fig S3A), that 77% of the centenarians had moderate-good vision (ESM.pdf Fig S3B), and that 80% of the centenarians were independently mobile (ESM.pdf Fig S3C). The majority (52%) of the centenarians in the cohort lived independently (i.e. community dwelling without assistance, or independent in a residence with available services), 42% lived in private quarters in a residential care center, while only 1.7% of the centenarians lived in a nursing home (ESM.pdf Fig S3D). Centenarians scored a median of 15 points (IQR 12–18), on the Barthel index: 45% of the centenarians scored between 15 and 19, which indicates a need for minimum help with activities of daily living (ADL), while 32% scored 20 points which indicates they are fully independent in ADL (ESM.pdf Fig S3E). The centenarians in the cohort have no or very few symptoms of depression: they scored a median of 2 points on the 15-items version of the Geriatric Depression Scale (IQR 1–3), and scores < 5 indicate no evidence for depression [29] (ESM.pdf Fig S3F).

### Disease prevalence and multi-morbidities

In June 2017 we had received GP reports from 209 centenarians, and categorized diagnosed conditions (Table 4). At baseline, centenarians were diagnosed with or had symptoms of on average 3.7 ± 1.5 morbidities (ESM.pdf Fig S3G). Cardiovascular problems are the most common condition in centenarians (83.7% has at least one mention of a cardiovascular condition in their GP report). And hypertension is mentioned in the GP reports of almost half of all centenarians. Removal of hypertension from the list of cardiovascular conditions still leaves 66.5% of the centenarians with at least one mention of a cardiovascular condition (Table 4). Musculoskeletal disease and hypertension were more prevalent in females (72 vs. 39% and 54 vs. 34%), while cardiovascular conditions were more prevalent in males (77 vs. 63%). Most aging-associated diseases were first mentioned in the GP report when the centenarian was > 90 years old, suggesting a seemingly high age at onset. As we cannot correct for methodological differences in data collection by GPs, we were not able to perform a systematic comparison with disease incidence statistics from prospective cohort studies (for further explanation see ‘age at disease onset’ analysis in ESM.pdf).

### Cognitive function (Mini Mental State Examination, MMSE)

**Fig. 5:**
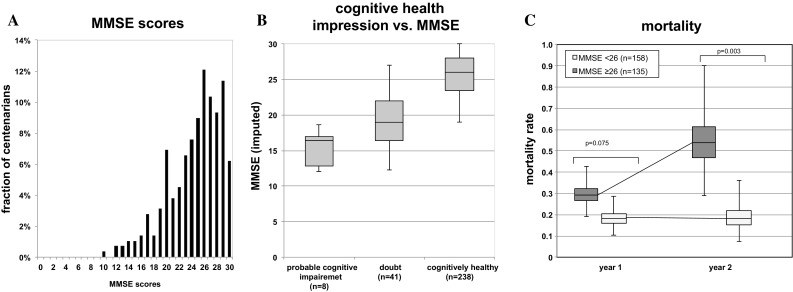
Overall cognitive functioning (Mini–Mental State Examination): **a** Mini–Mental State Examination (MMSE) scores. **b** Researcher impression of cognitive health at first visit, compared to MMSE score. **c** Mortality rate of centenarians with high and low performance on the MMSE

At cohort inclusion, the average raw MMSE score was 23.9 ± 4.4 points. We adjusted for missing items due to hearing or vision impairments, which allowed us to directly compare MMSE scores between centenarians (see Methods). At study inclusion the average adjusted MMSE score of the 100-plus Study cohort was 24.3 ± 4.23 points (median score 25, IQR 22.0–27.5) (Fig. 5a). For 287 centenarians, a trained researcher estimated cognitive health. The large majority (83%) of the centenarians was subjectively estimated to be cognitively healthy, and this group scored a median of 26 points on the MMSE (IQR 23.5–28). This was significantly higher than the median MMSE score of 19 (IQR 16.4–22) by the 41 centenarians for whom cognitive health was “doubted” (*p* = 4 × 10^−3^, two-tailed *t* test with unequal variance), and the median MMSE score of 8 centenarians who were estimated to have “probable cognitive impairment” was 16.4, (IQR 12.8–17) (Fig. 5b).

### MMSE and mortality rates

The mortality percentage (presented per annual-year) underestimates the mortality at extreme ages, such that we prefer presenting the instant mortality rates (presented per life-year); for rationale and calculation procedures see ESM.pdf. Within the group of 293 participants for which a baseline MMSE was available, there were 67 deaths that occurred before a next planned visit: the planning of a next visit was used to confirm which centenarians were still alive and who had died. There were 41 confirmed deaths that occurred before a planned first-year follow-up visit, and 174 centenarians were confirmed alive at the time of their first-year follow-up visit. The overall mortality rate in the first year after inclusion was 0.24 deaths per life-year (95% CI 0.17–0.32); which relates to a mortality percentage of 21% per annual year (95% CI 16–27%). Specifically, the 106 centenarians who scored ≥ 26 on the MMSE at baseline had a mortality rate of 0.19 deaths per life-year (95% CI 0.11–0.29), while the 109 centenarians with baseline MMSE scores < 26 had a mortality rate of 0.29 deaths per life-year (95% CI 0.19–0.43) (*p* = 0.075). Of the 91 centenarians who were eligible for a second follow-up visit, there were 20 confirmed deaths before this visit, and 71 were confirmed alive at the time of this visit. Therefore, in the second year after baseline, the mortality rate increased to 0.32 deaths per life-year (95% CI 0.20–0.49); which relates to a mortality percentage of 28% per annual-year (95% CI 18–39%). Specifically, the mortality rate of the centenarians who scored ≥ 26 points at baseline remained at a low 0.19 deaths per life-year (95% CI 0.08–0.37), while the mortality rate of centenarians who scored < 26 points increased to 0.54 deaths per life-year (95% CI 0.29–0.90) (*p* = 3.0 × 10^−3^) (Fig. 5c). Mortality rates and related mortality percentages are presented in Table 4.

### Education

We compared centenarian-education levels with individuals from the same birth cohort (1912–1916): 55–59 year-olds as reported in the Dutch population in the 1971 census [39]. Both centenarian-males and females attained significantly higher levels of education compared to their birth cohort in the 1971 census [33] (*p* < 1 × 10^−5^, Mann–Whitney U test) (ESM.pdf Fig S4A). Specifically, 79% of the centenarian males and 66% of the centenarian females attained more than basic education, compared to respectively 45% and 31% of the males and females in their birth cohort (Table 5). Workers and self-employed persons with little education were overrepresented in the ~ 20% non-responders in the 1971 census, suggesting that this is a conservative estimate of the differences [33].

### Socio-economic background and status

Based on *paternal* professions, centenarian—socio-economic background (SEB) was compared to 2815 individuals born between 1910 and 1915 from the Historical Sample of the Netherlands (HSN) [40] (*p* < 1 × 10^−5^, Mann–Whitney U test) (ESM.pdf Fig S4B-left). Centenarian-*fathers* were threefold more likely to have an elite-upper middle class occupation and > 3-fold less likely to be an unskilled worker compared to their birth cohort. Based on the professions of the 219 centenarian-males and centenarian-female-partners, centenarians *themselves* attained a significantly higher SES than the 408 males from the HSN sample born between 1910 and 1919 (*p* < 1 × 10^−5^, Mann–Whitney U test). Centenarians were > 4-fold more likely to be elite-upper middle class, > 2-fold more likely to be farmers, and > 3-fold less likely to be unskilled or farm workers (ESM.pdf Fig S4B-right). There was no difference between the socio-economic status (SES) attained as adults of 81 male centenarians and the male-partners of 138 female-centenarians (*p* = 0.22, Mann–Whitney U test).

### Smoking behavior and alcohol consumption

Retrospective comparison of smoking behavior suggests that centenarians smoked less than a representative sample of Dutch individuals born between 1909 and 1923, as indicated in a 1958 survey [41, 42]. Of the centenarian-males, 67% indicated to have smoked regularly or often during an extended period in their lives, while in 1958,  91% of the birth cohort males reported to smoke. Of the centenarian females, 15% indicated to have smoked regularly or often while 32% of the birth cohort females smoked. Alcohol consumption was common among centenarians: only 11% of the centenarian-males and 22% of the centenarian-females indicated to never consume alcohol, similar to 14% of male-abstainers and 21.8% female-abstainers among the birth cohorts in the 1958 survey [41]. In fact, 54% of the centenarian-males and 31% of the centenarian-females indicated to consume alcohol regularly or often.

### Marriage and children

Centenarians had on average 3.9 ± 2.2 children, which was more than the average 3.5 ± 2.5 children from 860 Dutch parents born between 1910 and 1915 [43] (*p* = 0.03, Mann–Whitney U) (ESM.pdf Fig S4C). However, we cannot exclude that lifestyle differences (i.e. religious or regional customs) might confound this increased fertility. Overall, 91% of the centenarians was ever-married, and 86% had one or more children. Of the centenarian females, 16.5% remained childless (36/219), similar to the 16% childless females born between 1915 and 1919 [44]. Five males in the cohort (6%) remained childless (birth cohort data not available [44]).

### APOE allele frequency

APOE was genotyped for 266 centenarians (ESM.pdf Fig S5). Centenarians were > 2-fold more likely to carry an *APOE*-ε2 allele than 2233 Dutch population controls aged 60–80 years [45]. Specifically, centenarians are 2.5-fold more likely to have the *APOE*-ε2/ε3 genotype (Table 5). In contrast, centenarians are > 2-fold *less* likely to carry an *APOE*-ε4 allele compared to the Dutch population; specifically, centenarians are 2.8-fold less likely to be genotyped *APOE*-ε3/ε4 and 6.7-fold less likely to have the *APOE*-ε4/ε4 genotype. The allele frequency of the *APOE* ε3 allele was identical for both cohorts.

## Discussion

Here, we present the 100-plus Study cohort of cognitively healthy centenarians based on the first 300 centenarians included in the 100-plus Study.

### On average, the centenarians in the 100-plus Study cohort have a high performance on the MMSE; the large majority is independent and retained hearing and vision abilities

Our inclusion criteria of “self-reported cognitive health, which is confirmed by an informant” led to a selection of centenarians with a relatively high level of overall cognitive functioning. The cohort scored an average 23.9 ± 4.4 points on the MMSE (raw, unimputed), which is considerably higher than the average MMSE score of ~17 points, by representative centenarian populations (16.2 ± 8.8 points, Georgia Centenarian Study [46]; 18.7 ± 7.4, Italian centenarians from Rome and surroundings [47]; 17.7 ± 8.3 centenarians from Northern Italy [48]). The overall cognitive performance of the 100-plus cohort participants is similar to “community-dwelling cognitively healthy centenarians” from the Georgia centenarian Study [49], and “cognitively healthy” Japanese centenarians, who respectively scored a mean of 24.8 points and 22.3 ± 3.32 points on the MMSE [50].

Next to their retained cognitive functioning, the large majority of the centenarians had moderate-good hearing and vision abilities, they were independently mobile, they enjoyed a relatively high level of independence in activities of daily living (ADL), and had no or few symptoms of depression. Centenarians were either community dwelling or lived independently in a residence or in a care center with available services. Together, these findings underscore that the 100-plus Study cohort is not a representative population of Dutch centenarians, rather, it represents a high-performing, independent sub-selection of Dutch centenarians.

### Cognitive performance is associated with mortality

The mortality in our cohort was 21% per annual year, which is two-fold lower relative to the centenarians in the general Dutch  population [51]. In the second year after baseline centenarians with high cognitive functioning at baseline retained a low mortality rate of 17% per annual-year, while centenarians with lower cognitive function at baseline had a mortality rate of 42% per annual-year. These findings confirm that we have succeeded in selecting the healthiest of the centenarians, as we assume that these will be maximally enriched with protective (genetic) factors. Our results further confirm that there is an overlapping etiology of maintained cognitive and overall health, and cognitive functioning [52, 53]. The longitudinal set-up of our study allows us to monitor changes in cognition in combination with other factors of overall health that occur between baseline and death to identify to which extent centenarians escaped or delayed cognitive impairment.

### The 100-plus cohort is twofold enriched with males

The fraction of centenarian-males is 27%, twice the fraction of males (14.4%) in the total Dutch centenarian population on January 1st 2017 [54]. Indeed, since dementia-prevalence in centenarian populations is consistently lower in males (~ 40%) than in females (~ 60%) [55, 56], we had on forehand expected that our inclusion criteria of “self-reported cognitive health, which is confirmed by an informant” might lead to a relative enrichment of centenarian males in the cohort. Based on the fraction of centenarian males in the population (14.3% in 2017) and lower dementia prevalence in males (40 vs. 60%), we estimated that the fraction of males in the 100-plus Study cohort should be approximately 20%. This suggests that dementia incidence does not fully explain the excess of males in our cohort, which leaves room for, for example, the influence of a participation bias and a better general well-being of centenarian males [57].

### Disease in male and female centenarians

Despite the cognitive health of the centenarians in the 100-plus Study cohort, they were diagnosed with on average four morbidities at baseline. Previous studies have shown that females are more prone to develop chronic nonfatal conditions such as dementia, arthritis and osteoporosis [58], while males are more likely to develop fatal conditions, such as cardiovascular disease and cancer [59, 60]. In agreement with these studies, we found that the females in the 100-plus Study cohort had a higher prevalence of musculoskeletal diseases and hypertension while males had a higher prevalence of heart disease and CVA/TIAs.

### The 100-plus Study cohort is equally depleted with the APOE-ε4 allele compared to other centenarian cohorts, but it is strongly enriched with the neuroprotective APOE-ε2 allele

The 100-plus Study cohort was 2.3-fold *less* likely to carry the *APOE-ε4* AD-risk allele compared to their birth cohort at 60–80-years (OR = 0.44, *p* = 6.3 × 10^−7^) [45]. This is in complete concordance with the depletion of the ε4 allele observed in a meta-analysis of 2776 (mostly Caucasian) centenarians and 12,000 controls (OR = 0.43, *p* < 1 × 10^−3^) [61]. It is well established that carrying one or two *APOE*-ε4 alleles is associated with respectively a 3–5 and 10–30-fold *increased* risk of developing AD. Depletion of the *APOE*-ε4 allele in centenarians confirms that ε4 allele carriers dropped out of the population during aging [62]. On the other hand, carrying one protective *APOE*-ε2 allele is associated with a two-fold *decreased* lifetime risk of developing AD [63, 64]. But the large centenarian meta-analysis indicated that the protective aspect of the *APOE*-ε2 allele does not extend to an enrichment in centenarians (OR = 1.08, *p* = 0.66), although weak evidence for an enrichment of the *APOE*-ε2/ε3 genotype was observed (OR = 1.4, *p* = 1.7 × 10^−2^) [61]. In contrast, we find strong evidence for an enrichment of the *APOE-ε2 *allele in cognitively healthy centenarians from the 100-plus Study cohort compared to their birth cohort at 60–80-years: centenarians were 2.1-fold more likely to carry the *APOE*-ε2 allele (*p* = 4.8 × 10^−7^), and 2.5-fold more likely to have an *APOE*-ε2/ε3 genotype (*p* = 3.4 × 10^−7^). This confirms previous suggestive findings in a cohort of Italian centenarians who were free of dementia or any other major age-related conditions, which had a similar enrichment of the *APOE*-*ε2* allele [65]. We speculate that this enrichment of the *APOE*-ε2 allele is not a consequence of our selection of extreme ages, but that it reflects our selection of individuals with retained (cognitive) health until extreme ages.

The specific enrichment of the *APOE*-*ε2* in the centenarians with high cognitive performance suggests that the etiology for reaching 100 years with maintained (cognitive) health may be distinct from the etiology of reaching 100 years in general. Our results indicate that while searching for (genetic) factors that maintain cognitive health, the *APOE* genotype should be taken into account.

### Centenarians came, on average, from higher socio-economic classes and had higher levels of education

On average, centenarians came from a higher socio-economic background than their birth cohort. A high fraction of centenarian-fathers were farmers, mostly on their own farm, a common occupation in the Netherlands during the early twentieth century. As adults, centenarians attained a higher socio-economic status and they had more children compared to their birth cohort. Both male- and female-centenarians attained higher levels of education than the males and females from their birth cohorts. These findings reflect the selective survival advantage of individuals from the higher/middle socioeconomic classes and farmers, during the majority of the twentieth century in the Netherlands [66]. Together, this is in agreement with results from several centenarian studies, which showed that socioeconomic background, educational attainment, and adult socioeconomic status influenced the chance to become a centenarian [67]. Likewise, having children associates with an increased chance of reaching extreme ages, likely due to the involvement of children in the care for their aged parent [68].

### Alcohol consumption of centenarians was similar to birth cohort peers, and they smoked—but less

Two-thirds of the centenarian males and 15% of the centenarian females indicated to have smoked regularly or often during an extended period in their life. This was less than their birth cohort peers, of whom almost all males and a third of the females smoked [41]. Alcohol consumption of the centenarians was similar to their birth cohorts. These results are partly in agreement with lifestyle behaviours from the American Ashkenazi Jewish centenarians, whose alcohol consumption and smoking behaviour was not different from the general population [69].

We note that comparisons of lifestyle habits such as alcohol consumption and smoking rely on recall of habits from several decades ago, which may introduce recall bias. For this reason, we focused on investigating lifestyle factors that are manifest for a longer period during a lifetime. Habits that may be more variable throughout life, such as dietary or exercise habits, might be more difficult to recall and we chose to refrain from investigating these. Despite these limitations, a within-cohort analysis of these variables may add to the rich phenotypic data available for this cohort.

## Conclusions

The 100-plus Study cohort represents cognitively healthy Dutch centenarians. Compared to their birth cohort peers, centenarians from this cohort attained significantly higher levels of education, were from a higher socioeconomic background, attained higher socioeconomic status, and they had more children, all of which confirms previous findings that these factors are associated with the chance of reaching 100 years in cognitive health. The combined contributions of these features, which are often concentrated within families, and the enrichment with the genetically heritable *APOE*-*ε2* allele, will most likely explain a considerable proportion of the high heritability of reaching 100 years with maintained cognitive health. However, these features do not apply to all centenarians, and only a third of the cohort carries the *APOE*-*ε2* allele. This suggests that additional protective factors may account for the cohort phenotype.

With the recent developments in biotechnology, novel findings regarding the physiology of exceptional longevity and cognitive function are emerging [70, 71]. The availability of blood and brain tissues from the healthiest centenarians provides the opportunity to acquire insights in the molecular constellations associated with the long-term maintenance of cognitive health. Ultimately, with this cohort we aim to contribute to the generation of novel hypotheses regarding the generation of novel therapeutic targets that offer resilience to cognitive decline.

### Electronic supplementary material

Below is the link to the electronic supplementary material.

Supplementary material 1 (DOCX 5685 kb)

## Figures and Tables

**Figure:**
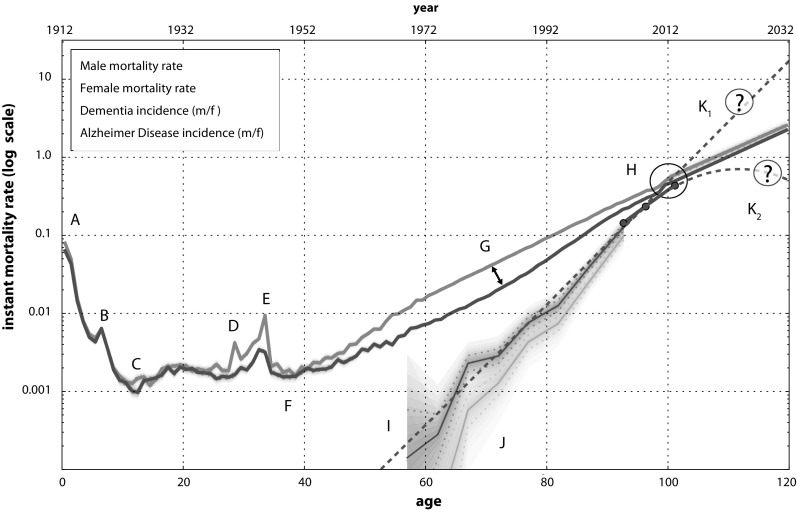
**Box-Figure: Instant mortality and dementia rates in centenarian birth cohort**. Blue line: Male mortality. Shades of blue represent confidence intervals (CI) on the mortality rate by 10-percentile increments [84]; for ages 0–59 years we used only the mortality statistics of individuals born in 1912 (as to avoid blurring specific mortality peaks), and for ages 60–100 years we combined statistics of the 1910–1915 birth cohorts, which reduced CIs. Mortality after age 100 years was extrapolated in accordance with the Gompertz’ law of mortality [85]. Purple line: Female mortality with CIs [84]. Red line: Median incidence of overall dementia with CIs for age groups 55–59, 60–64, 65–65, 70–74, 75–79, 80–84, 85–89 years [1]. To define mean age per age-group, we assumed that the ages of the individuals that constituted each age-group were distributed according to associated mortality statistics. Red dots: Dementia incidence for age groups: 90–94 years (mean age 92.7), 95–99 years (mean age 96.4) and 100 + (mean age 101.3) [2]. Green line: Alzheimer’s Disease (AD) incidence with CIs [1]. Dashed red line: extrapolation of dementia incidence according to its exponential increase. To extrapolate dementia incidence, we fitted a Gompertz curve on available dementia incidence data [1, 2]. For the reported age ranges we compared the estimated dementia incidence with the reported incidence through a binomial distribution. This resulted in a log-likelihood, which was optimized (see ESM.pdf for mortality calculations)

## Box: Study rationale

**Table:**

The design of an intervention for neurodegenerative diseases requires not only the understanding of the neurodegenerative processes involved, but also a deep comprehension of the processes that *maintain* cognitive health during ageing. Although increasing age is the strongest predictive factor for cognitive decline and dementia, some people live to be over 110 years in great mental health. A Dutch woman, Hendrikje van Andel-Schipper (1890–2005), reached the age of 115 with full cognitive abilities [3] and showed that *it is possible to reach extreme ages without any symptoms of cognitive decline.* Her remarkable case became the source of inspiration for the initiation of the 100-plus Study at the Alzheimer Center Amsterdam in 2013. To investigate the physiology of her extended cognitive health, it is necessary to compare her clinical characteristics with those from other individuals with the same extraordinary combination of phenotypes: extremely old and cognitively healthy. Below, we provide a rationale for researching protective factors against cognitive decline in cognitively healthy centenarians, based on the mortality rate and dementia incidence in their birth cohort during its process of aging.
_____
The number of centenarians in the Netherlands is growing quickly: on January 1st 2013 there were 1940 centenarians in the Netherlands, which grew to 2225 centenarians on January 1st 2017, and this number is expected to rise to 5000 by 2035 [54]. Of the individuals born between 1910 and 1915, approximately 1:160 (0.6%) have reached ages ≥ 100 years [72]. Since 25–30% of all centenarians are estimated to be free of symptoms of cognitive decline [74–76], becoming a centenarian with retained cognitive health was reserved for only 0.2–0.3% of the 1910 and 1915 birth generation.
Almost all participants of the 100-plus Study cohort were born in the Netherlands just before or during WWI (1914 and 1917), in which the Netherlands was neutral. The 20th century in the Netherlands was further characterized by a depression in the thirties, WWII between 1940 and 1945, a post-war period typified by a rebuilding phase in the 1950s and a continuous increase in prosperity, health care improvements and technological developments. According to the Human Mortality Database [72] males born in the Netherlands between 1910 and 1915 had a mean lifespan of 58.7 years and females had a mean lifespan of 66.1 years.
Here we describe the 1910–1915 birth cohort by their mortality rates and dementia incidence from birth to > 100 years (Box-Figure). For this, we prefer presenting the instant mortality rate over the mortality percentage, because, while estimates are similar at younger ages, the mortality percentage underestimates the mortality at extreme ages (for further explanation see Mortality estimations in ESM.pdf). For ages 0–60 years, we represent mortality rate by age from individuals born in 1912, and for ages > 60 years we represent mortality rates using combined statistics from the 1910–1915 birth cohorts.
(**A**) In 1912, 170,000 babies were born, and they were exposed to a mortality rate of close to 0.10 deaths life-year (~ 10% per annual-year) during their first year of life, reflecting prevalent childhood diseases. (**B**) In 1918, the Spanish Flu made > 40,000 casualties in the Netherlands [77], which was especially lethal among 20–40 year olds [78]. The Spanish Flu increased mortality among the 1912-born six-year olds by twofold. (**C**) At age 10, most childhood diseases were overcome and the mortality rate reached a stable ~ 0.001–0.002 deaths per life-year (~ 0.10–0.20% per annual-year), caused by incidental deaths (i.e. fatal accidents, drowning etc. (**D**) When the 1912 birth cohort was 28 years, the onset of WWII in 1940 led to a peak in male mortality. (**E**) The ensuing Hunger Winter in 1945 led to a second mortality peak when the 1912 birth cohort was 33 years old, more so in males than in females. (**F**) When the cohort was 40 years old, the mortality resulting from natural decline rose above the rate of incidental deaths, and increased in the log scale according to Gompertz Law (1825) [79]. (**G**) During natural decline of the 1912 birth cohort, the males had a higher mortality rate than females, and this mortality gender gap ultimately resulted in a 1:7 male/female ratio at age 100 [54]. Approximately 70% of the mortality gender gap in these cohorts can be explained by the difference in smoking behavior between males and females [80]: an estimated 91% of all males born in 1912 smoked while only 30% of the females smoked, which ultimately led to a relative increased incidence of fatal smoking-related diseases in males. The remainder of the mortality gender gap may be explained by biological or environmental differences between males and females [81]. (**H**) At 100 years old, the 1912 cohort has reduced to ± 1000 persons and the instant mortality rate for both males and females is at 0.5 deaths per life-year (which translates to a mortality percentage of ~ 40% per annual-year).
(**I**) Individuals from the 1910–1915 birth cohorts were exposed to an increasing incidence of overall dementia from age 60 years onwards, of which the greatest proportion was (**J**) Alzheimer’s Dementia (AD) [1]. At approximately 100 years, the instant incidence of dementia reaches 0.5 cases per dementia-free year, which translates to a dementia proportion of ~ 40% per annual-year. At this age, the dementia incidence surpasses the mortality rate per year, suggesting that after turning 100 years, a centenarian is exposed to greater odds of developing dementia than to die [6]. (**K**_**1**_) If dementia incidence after 100 years continues to increase exponentially, following the Gompertz law of natural decline [79], then a conservative estimation of dementia incidence (by concentrating mortality on incident dementia cases) suggests that all individuals who reach 108–110 years would have to be demented. (**K**_**2**_) In contrast, reports of individuals who are older than 110 years indicate that the majority of such individuals has, in fact, retained their cognitive health [3–5]. Therefore, it is likely that the incidence of dementia decelerates or even declines at extreme ages [6]. Although the slope of the incidence rate suggested by Corrada et al. (red dots in Box-Figure) is slightly smaller compared to the extrapolated incidence (dashed line), there currently is no clear evidence for this deceleration between 90 and 100 years [2, 82], it is most likely that this deceleration becomes evident somewhere after 100 years. This is consistent with findings in super-centenarians by Andersen et al. [5], who demonstrated the progressive compression of both disability and morbidity (in 6 diseases including dementia) with survival beyond 100 years. Furthermore, in a recent study based on data from 3836 centenarians in Italy, Barbi et al. found that mortality decelerates, and even plateaus, above age 105 [83]. Together, this suggests that factors that preserve (cognitive) health may be progressively enriched for during healthy aging [7], providing a window of opportunity to search for such protective factors in a population of healthy (super-)centenarians.
