# Supplementary material for: The 100-plus Study of cognitively healthy centenarians: rationale, design and cohort description
Source: Eur J Epidemiol. 2018 Oct 25;33(12):1229–49. doi: 10.1007/s10654-018-0451-3 (PMC6290855; doi:10.1007/s10654-018-0451-3)
Supplement: Supplementary file 1 — Supplementary material 1 (DOCX 5685 kb) [file 10654_2018_451_MOESM1_ESM.docx]

**SUPPLEMENTARY MATERIAL:**

**The 100-plus Study of cognitively healthy centenarians: rationale, design and cohort description**

**Authors:** Henne Holstege PhD^1,2,#^, Nina Beker MSc^1^, Tjitske Dijkstra MD^1^, Karlijn Pieterse MSc^1^, Elizabeth Wemmenhove MSc^1^, Kimja Schouten^2^, Linette Thiessens MSc^1^, Debbie Horsten MSc^1^, Sterre Rechtuijt MSc^1^, Sietske Sikkes PhD^1^, Frans W.A. van Poppel PhD^3^, Hanne Meijers-Heijboer PhD^2^, Marc Hulsman PhD^1,2^, Philip Scheltens MD PhD^1^

**Author affiliations:** ^1^Alzheimer Center Amsterdam, Department of Neurology, Amsterdam Neuroscience, Vrije Universiteit Amsterdam, Amsterdam UMC, de Boelelaan 1118, 1081 HZ Amsterdam, The Netherlands; ^2^Department of Clinical Genetics, Amsterdam Neuroscience, Vrije Universiteit Amsterdam, Amsterdam UMC, de Boelelaan 1118, 1081 HZ Amsterdam, The Netherlands; ^3^Netherlands Interdisciplinary Demographic Institute (NIDI/KNAW), Lange Houtstraat 19. 2511 CV The Hague, The Netherlands

# Corresponding Author

Dr. Henne Holstege

Alzheimer Center Amsterdam, Amsterdam UMC,

de Boelelaan 1118

1081 HZ Amsterdam, The Netherlands

Tel: +31 20 4440816

Fax: +31 20 4448529

Email: h.holstege@vumc.nl

Table of Contents

Participant recruitment and contact 4

Recruitment 4

Informed consent 4

Contact with centenarians and family members 5

Mortality estimation 5

MMSE imputation 7

Age at disease onset analysis 8

100-plus Study variables 9

Demographic/lifestyle characteristics 9

Gender 9

Year of birth 9

Age 9

Death 9

Visits 9

Lifetime history (self-report) 9

Family genealogy and disease occurrence 9

BMI during adulthood 10

Level of education 10

Social Economic Status (SES) 10

Social Economic Background (SEB) 11

Smoking 11

Alcohol consumption 11

Baseline performance 12

MMSE 12

Imputed MMSE scores 12

Cognitive functioning (subjective) 12

Follow-up of Cognitive functioning: telephone and mail 12

Cognitive health (objective) 12

Continence 12

Comorbidities 12

Medical History (self report) 12

Medical History GP report 13

Oral health condition 13

Independent living 13

Vision 13

Hearing 13

Mobility 13

Activities of daily living (ADL) 14

Total hours of care per week 14

Blood pressure and heartbeat 14

Grip Strength 14

Sleeping habits 14

Collected Biomaterials 15

Blood sample 15

Genetic: WES 15

Genetic: GWAS 15

Genetic: APOE 15

Post-mortem brain donation 15

PET-MRI or PET-CT brain imaging 15

Gut Microbiome 16

Supplementary Figures 17

Fig. S1 Demographics of first 300 centenarians from 100-plus Study cohort 17

Fig S2 Catchment Area by birth municipality. 18

Fig S3 Baseline presentation of centenarians 19

Fig S4 Lifestyle and socioeconomic characteristics of centenarians 20

Fig S5 APOE allele distribution of centenarians in 100-plus Study cohort 21

References 22

# Participant recruitment and contact

## Recruitment

Using pre-defined queries we regularly perform an online search for local newspaper-reports, which mention a centenarian. For example, it is custom in the Netherlands that when individuals celebrate their 100^th^ birthday, the local mayor visits. Articles that mention a centenarian commonly include the name of the centenarian, and in some cases a description of their well-being, and the city and care-center where the centenarian lives. The study has also become well-known in the Netherlands such that in some cases centenarians or their proxies contact us to inform about possibilities for study participation.

After retrieving a contact address we send a letter to the prospective study participant in which we introduce the study, its objective and its procedures. We also include the study information folder (Dutch, downloadable at www.100plus.nl). After receiving this information, prospective study participants either call us to express their interest in study participation, or we approach the centenarian candidate and/or their family members within two weeks. If the centenarian agrees with study enrolment, and inclusion criteria are met, we schedule two baseline visits. In a letter we confirm visit-dates and –times and we describe the visit-procedures. Also, to ensure that all those involved feel maximally at ease about study participation, we indicate that we appreciate the presence of at least one proxy during the visits. Once a centenarian has agreed to study participation, their siblings, children and respective partners are eligible for study inclusion.

## Informed consent

With the letter confirming study inclusion we send the informed consent form (ICF). For all participants, formal consent for study participation is acquired during the first meeting with a study-researcher. For centenarians, age is verified by an official document stating the birth date, (birth certificate, a driver’s license, or an ID card). In case visual disabilities or trembling complicate reading and signing the ICF we ask a proxy to read the ICF out loud. If the centenarian is unable to sign, the proxy signs while stating his/her relationship with the centenarian. The ICF includes the consent to notify the GP of study participation, which involves a request of medical history. Also, the ICF includes the consent to donation of a blood sample, and the opt-in for the generation of induced pluripotent stem cells (iPSC’s) from the blood sample. We ask consent to approach the GP after the death of participant to request the medical events that preceded death.

## Contact with centenarians and family members

We inform participants about the study proceedings with an annual newsletter, during an annual 100-plus Study participants Day and a Christmas/New Year’s greeting card. We host a website ([www.100plus.nl](http://www.100plus.nl)) which includes information about the 100-plus Study: study procedures, study rationale, events that are organized for the centenarians, and newsworthy items.

# Mortality estimation

**Estimation of mortality rates in 100-plus Study cohort:** For the estimation of mortality in the 100-plus Study cohort, we report the instant mortality rate *_n_M_x_*, which considers the exact time each person is alive in the study, and is expressed in person-time [[1](#_ENREF_1)]:

***mortality rate: _n_M_x_ = _n_d_x_ / _n_L_x_,***

where *x* is the years after study inclusion, *n* is the time interval considered (in years), *_n_d_x_* is the number of deaths during the time interval between *x* and *x+n*, and *_n_L_x_,* is the time each person was alive during that time interval, totaled for all persons.

To calculate the mortality rate, we take only deaths into account that occur before a potential yearly visit, such that all deaths are verified during the planning of a follow-up visit, and we consider the time between potential yearly visits. For living participants and participants who died after the last visit, we right-censored after the last visit. Survival times were calculated using exact dates. Mortality rates were estimated based on survival curves constructed using the Beta Product Confidence Procedure (BPCP), which provides stable confidence intervals on the survival curves for small samples or heavy censoring [[2](#_ENREF_2)].

**Evaluation of mortality in the 1910 – 1916 birth cohorts (Box-Figure in main document):** For each birth cohort, the age-specific mortality proportion ***_n_q_x_*** can be directly derived from the population life-tables [[3](#_ENREF_3)]:

***mortality proportion at age x: _n_q_x_ = _n_d_x_ / _n_l_X_***

where *x* is the age in years, ***_n_d_x_*** is the number of deaths that occurred during the year in which individuals were aged *x,* and *_n_l_X_ is* the number alive at age *x*. As we determine the mortality proportion during the year individuals have age *x*, the time interval considered (*n)* is 1 year. However, at extreme ages, the mortality proportion does not properly represent the true mortality. This can be observed by noting the difference between calculations for mortality rate and proportion presented above: i.e. between *_n_L_x_,* and *_n_l_x_. _n_L_x_* represents the totaled life-time of the population during the year (the period after a death occurs does not count towards this life-time). In contrast, *_n_l_X_* represents the population size at the beginning of the year, and can therefore also be used as the totaled life-time of the population during the ensuing year, assuming negligible death. For ages less than 90 years, *_n_l_X_* and *_n_L_x_* approximate each other, as only a small fraction of the population dies during the year. However, for ages >90 years there is a marked reduction in the population between age *x* and *x+*1, due to which *_n_L_x_* is significantly lower than *_n_I_x._* Therefore, at extreme ages, the use of the mortality proportion leads to an underestimation of the true mortality at extreme ages, falsely suggesting a deceleration of mortality at extreme ages (see Figure below for the 1912 birth cohort). Note also that the age-specific mortality proportion is restricted and will converge to 100%, while mortality rate theoretically can grow to infinity. Indeed, the Gompertz law of mortality suggests the age-specific mortality rate increases exponentially with age, while the age-specific mortality proportion does not [[5](#_ENREF_5), [6](#_ENREF_6)]. To indicate the different representation of mortality, we express mortality rate as ‘per life-year’ and mortality proportion as ‘per annual-year’.

The age-specific mortality rate (*_n_M_x_*) can be derived from the mortality proportion (*_n_q_x_*) using a simple method suggested by Fergany [[4](#_ENREF_4)], which is based on the exponential distribution.

***mortality rate at age x: _n_M_x_ = -(log(1 – _n_q_x_))/n***

Age-specific mortality rates can be transformed into age-specific mortality proportions by the reverse relationship:

***mortality proportion at age x: _n_q_x_ = 1 – exp(–n*_n_M_x_ )***

In the Box-Figure in the main document we determined confidence intervals for the mortality rate using a Monte Carlo procedure, in which samples were obtained from the Beta Product distribution, converted to mortality rates, and used to estimate distribution percentiles.

***Figure:*** *Age-specific mortality proportions were determined using life-tables from the 1912 birth cohort (available until age 98), and these were transformed into age-specific instant mortality rates. In accordance with Makeham-Gompertz law of mortality, the mortality rates increased exponentially with age after the cohort reached ~40 years. Mortality rates after 98 years were determined by extrapolation using the slope of this increase (determined based on the mortality rates between 50 and 98 years). The extrapolated mortality rates were then transformed into the mortality proportions after age 98, such that mortality proportions could also be extrapolated to extreme ages. As indicated in this example of the 1912 birth cohort, mortality rates will always be higher than the proportions: at lower ages the difference between mortality proportion and the mortality rate is negligible, but at higher ages the former will converge 100%, while the latter will continue to increase.*

# MMSE imputation

When items in the Mini Mental State Examination could not be scored due to vision or hearing impairments, the total obtainable score is different per centenarian. Therefore, raw MMSE scores that include missing items cannot be directly compared among centenarians. To address this, we adjust scores using multiple imputation by chained equations using 10 iterations (MICE R-package [[7](#_ENREF_7)]), provided that the number of missing items represented no more than 6 points on the total MMSE score. Variables used for imputation: MMSE scores per item, the subjective researcher-estimate of hearing and visual abilities, subjective impression of cognitive health, gender and educational level. We averaged imputed MMSE scores across 25 imputations.

**Effect of imputation on MMSE scores of the first 300 centenarians entered in the study:** 69% of the centenarians (n=206) did not miss any items on the MMSE, and these scores were not imputed. Imputation increased MMSE scores on average 0.7±0.2 points for those who missed one item (n=36), an average 1.4±0.3 points for those who missed two items (n=21), an average 2.3±0.3 points for those who missed three items (n=14), an average 2.7±0.5 points for those who missed four items (n=5), 3.6±0.9 points for those who missed five items (n=6), and an average 4.3±1.6 points for the centenarians who missed 6 items (n=2). 3% of the centenarians (n=10) had too many missing items and these scores were not adjusted by imputation and excluded from analysis.

# Age at disease onset analysis

The median age at which a disease was first mentioned in the GP reports was 93 years for hypertension (IQR: 87 - 97), 96.5 years for cardiovascular disease (IQR: 93 - 98), 94 years for arthrosis (IQR: 86 - 97); 94.5 years for skin cancer (IQR: 92.75 - 97), and these age ranges were similar for almost all other diseases. However, entries in GP records may be biased towards diagnoses given after digitalization of GP records during the last ~25 years. Furthermore, technical advances in medical practice and increased attention for prevention of specific diseases may influence disease diagnosis and thus confound disease incidence statistics. We cannot correct for such confounders in this retrospective analysis, and therefore we refrained from performing a systematic comparison of these data with age at onset statistics derived from prospective cohort studies.

# 100-plus Study variables

**CEN:** Data collected from centenarians

**CP:** Data collected from children of centenarians and their partners

**SP:** Data collected from siblings of centenarians and their partners

| Demographic/lifestyle characteristics | |
| --- | --- |
| Gender **CEN, SP, CP** | 1: Male  2: Female |
| Year of birth **CEN, SP, CP** | Birth year (due to privacy considerations we do not store birth dates) |
| Age **CEN, SP, CP** | Age on date of baseline visit |
| Death **CEN, SP** | We ask informants to inform us when a participant dies. Deaths that were not reported become apparent during the planning of a new visit.   - Age at death - Cause of death - Brain donor (yes/no, autopsy date) - Process of death: acute death/slow decline, involvement of disease, palliative sedation, euthanasia, reduced food/fluid intake, external event, cognitive health until death |
| Visits **CEN, SP, CP** | - Date of baseline visit 1 - Date of baseline visit 2 - Date(s) of follow-up visit(s) |
| Lifetime history (self-report) **CEN** | During the baseline visit, we inquire about**:**   - Birthplace - Main youth living environment (countryside, seaside, city environment) - Religion - Marital status - Year of marriage - When applicable, year of divorce - WWII Camp survivor - Famine during Hunger Winter (WWII) - Cause of death of partner - Age of death partner - Number of children - Number of siblings - Number of paternal aunts and uncles - Number of maternal aunts and uncles - Number of partners - Year of birth of last child - Age of retirement - Age of when the centenarian actively contributed to society, e.g. by doing volunteer work or holding management/administrative functions. |
| Family genealogy and disease occurrence **CEN, SP, CP** | We draw a pedigree including children, siblings, parents and grandparents: we record:   - (Maiden) names - Gender - Birth years - Age at death and cause of death - Wedding date(s), and if applicable divorce date(s)   We ask at what age family members had a formal dementia diagnosis or showed signs of cognitive decline, diabetes, TIA, stroke (CVA), heart disease, heart attack, cancer, Parkinson's disease, and whether symptoms occurred before or after the 70^th^ year of life. We ask children and the siblings of centenarians and their respective partners about birth years, age of death and dementia symptoms and diagnoses of family members. |
| BMI during adulthood **CEN, SP** | - Self-reported weight at middle-age (~50 years) - Self-reported length at middle age (~50 years)   BMI is categorized according to common standards: underweight (<18), normal weight (18-25), overweight (25-30) or obese (>30).  We further ask whether the centenarian lost weight during the hunger winter of 1944-1945 or during time spent in concentration camps.  We inquire whether the centenarian ever experienced weight fluctuations of >10 kg within one year (pregnancies excluded) and for both the centenarians and their siblings we ask whether they dropped >5 kg in the past year. |
| Level of education **CEN, SP, CP** | - Years of education - Education level, categorized in: - Verhage education level [[8](#_ENREF_8)]  1. Did not finish primary school 2. Finished primary school 3. Did not finish secondary school 4. Finished secondary school, low level 5. Finished secondary school, medium level 6. Finished secondary school, highest level, and/or college degree 7. University degree.  - The ISCED 1997 classification includes 7 categories [[9](#_ENREF_9)]:  1. Pre-primary education 2. Primary education or first stage of basic education 3. Lower secondary or second stage of basic education 4. (Upper) secondary education 5. Post-secondary non-tertiary education 6. First stage of tertiary education 7. Second stage of tertiary education  - The education levels in the Dutch 1971 census education are [[10](#_ENREF_10)]:  1. Basic or less (primary school or less) 2. Low (1 to 2 years secondary (unfinished) 3. Extended low (3 to 4 years secondary, or lower technical) 4. Medium (5 to 6 years secondary or medium technical/vocational) 5. Semi-high (applied sciences or first year university) 6. High (university or alike) |
| Social Economic Status (SES) **CEN, SP, CP** | We inquire about the main occupation and/or the highest-level occupation of the centenarian, their parents and their partner(s).   - Profession centenarian - Profession father - Profession mother - Profession partner   Occupations are classified according to the coding scheme of the Historical International Standard Classification of Occupations (HISCO)[ [11](#_ENREF_11), [12](#_ENREF_12)]. HISCO occupation codes are classified into social classes according to the HISCLASS-5 scheme, a commonly used 5-class version of the HISCLASS system[[13](#_ENREF_13)]. Since centenarian-females were mostly housewives, we determined their SES based on the occupations of their spouses. Profession category: HISCLASS-5 score: based on occupation of centenarian or centenarian-partner:   1. Unskilled workers and farm workers. 2. Skilled workers, medium skilled and lower skilled; 3. Self-employed farmers and fishermen; 4. Lower middle class, lower managers, professionals, clerical and sales personnel and foremen; 5. Elite and upper middle class, nobility, higher managers and higher professionals |
| Social Economic Background (SEB) **CEN, SP, CP** | HISCLASS-5 score: based on occupation of centenarian-father   1. Unskilled workers and farm workers. 2. Skilled workers, medium skilled and lower skilled; 3. Self-employed farmers and fishermen; 4. Lower middle class, lower managers, professionals, clerical and sales personnel and foremen; 5. Elite and upper middle class, nobility, higher managers and higher professionals |
| Smoking **CEN, SP, CP** | - Smoking behaviors **(**packs per week, per decade); We ask whether the centenarian:  1. Currently smokes 2. Never smoked 3. Used to smoke but not anymore  - We ask how much the centenarian smoked:  1. Never: has never smoked 2. Rarely: has sporadically smoked a cigarette, never on regular basis 3. Occasionally: i.e. at a special occasion 4. Regularly: regularly but not more than 2 packs per week 5. Often: >2 packs per week  - We ask whether the centenarian smoked when they were aged:  1. 0-10 years 2. 10-20 years 3. 20-30 years 4. ..., 5. 90-100 years  - We ask if the centenarian was regularly exposed to passive smoking during their lifetime (yes/no). |
| Alcohol consumption **CEN, SP, CP** | - Current alcohol consumption (per glasses per week, per decade). We ask whether the centenarian:  1. Currently drinks alcoholic beverages 2. Abstained from alcohol or 3. Used to drink alcohol but not anymore.  - Lifetime alcohol consumption (per glasses per week, per decade)  1. Never: never drank alcohol 2. Rarely: now and then at a special occasion. 3. Occasionally: i.e. at a party, no more than several (2-4) glasses per month 4. Regularly: several (2-4) glasses per week; occasionally 5. Often: drank almost every day (≥1 glasses of alcohol per day ≥5 days per week)  - Age of alcohol consumption: We ask whether the centenarian drank alcoholic beverages when they were aged:  1. 0-10 years 2. 10-20 years 3. 20-30 years 4. ..., 5. 90-100 years |

| Baseline performance | |
| --- | --- |
| MMSE **CEN, SP** | Overall cognitive functioning was measured by the Mini-Mental State Examination [[14](#_ENREF_14), [15](#_ENREF_15)], maximum 30 points, scores were stored at the item level. Interpretation according to Tombaugh and McIntyre 1992[[16](#_ENREF_16)] |
| Imputed MMSE scores **CEN, SP** | When geriatric sensory impairments led to missing items on the MMSE we adjust MMSE scores using multiple imputation (see MMSE imputation) |
| Cognitive functioning (subjective) **CEN, SP** | Researchers are trained to recognize when participants:   - Continually repeat themselves - Are fatigued/doze off - Have difficulties with naming/word finding - Have difficulties recollecting important life-events - Have difficulties with understanding or remembering test assignments or interview questions   Cognitive health of participants is subjectively estimated:   - Cognitively healthy: <2 of the features apply - Doubt: 2-3 of the features apply - Probably cognitively impaired: >3 of the features applies |
| Follow-up of Cognitive functioning: telephone and mail **CEN, SP** | For half yearly follow up of cognitive health for brain donors, for follow-up of centenarian siblings or when MMSE of a centenarian at last visit dropped ≤20 points we use:   - Informant Questionnaire on Cognitive Decline in the Elderly, Dutch version (IQ-CODE-N, by mail) [[17](#_ENREF_17), [18](#_ENREF_18)]. - TICs-M (by telephone) [[19](#_ENREF_19)] |
| Cognitive health (objective) **CEN, SP*** | Neuropsychological test battery (**Table 2**, main manuscript)  Covers overall cognitive functioning (MMSE), memory, pre-morbid intelligence, language, attention and/or concentration, executive and visuo-spatial functions.  *MMSE only |
| Continence **CEN** | We ask whether the centenarian suffers from incontinence and when symptoms started.   1. No (or with catheter) 2. Yes, Incontinence started:   1 year ago  1-5 years ago  5-10 years ago  10-20 years ago  > 20 years ago |
| Comorbidities **CEN** | For each centenarian we obtained a **General Practitioner summary report**, which lists diagnosed conditions and prescribed medications. For each centenarian we determined whether they had conditions that belonged to each of the disease-categories listed in **Table 4** (main manuscript). Then we determined the number of unique disease-categories applied to each centenarian. |
| Medical History (self report) **CEN, SP, CP** | Centenarians:   - Complications during birth - Childhood health issues (other than common childhood diseases) - Spanish Flu in 1918 - Age at menarche (females only) - Onset of menopause/last menstruation (females only) - Number of full term pregnancies (females only) - Number of miscarriages (females only) - Hospital visits (age, cause) - Surgeries (age, cause) - Anesthetics (age, cause) - We document since what age the centenarian is familiar with:   High blood pressure  Heart disease  Stroke (CVA) or TIA  Tumor  Head injury  Mental health problems  Active infections  Children/siblings and their partners:   - history of high blood pressure, diabetes, heart disease, osteoporosis, stroke (CVA) or TIA; tumor, auto-immune diseases, active infections, and medication intake. |
| Medical History GP report **CEN** | - Prescribed medications: cardiac, diuretics, anticoagulants, laxatives, antacid, sedatives, anti-diabetics, antidepressants, iron, other - Vitamins |
| Oral health condition **CEN (subset)** | - Preservation of natural teeth, pain and/or discomfort, chewing ability, and xerostomia, and date of last visit to an oral health care provider (collection stopped in 2015). |
| **Depression**  **CEN** | - 15-item Geriatric Depression Scale (GDS-15) [[20](#_ENREF_20), [21](#_ENREF_21)] score range 0-15;  1. ≤5 points: no evidence for depression, 2. >5 points: indication for depression and warrants follow-up. |
| Independent living **CEN, SP** | - Current residence type  1. In a nursing home 2. Full time assistance at home residence 3. Private quarters in a residential care center 4. With a child or family member 5. community dwelling or in a private residence with services  - In case of dependent living, reason(s) for moving to a care center or nursing home - Age of living in residence - Age of leaving residence - Support medical care - Support meal service - Support household help (stopped) |
| Vision **CEN, SP** | - Subjective Researcher Impression:  1. Very poor 2. Poor 3. Moderate 4. Good |
| Hearing **CEN, SP** | - Subjective Researcher Impression:  1. Very poor 2. Poor 3. Moderate 4. Good |
| Mobility **CEN, SP** | - Subjective Researcher Impression:  1. Cannot move independently 2. Can move around independently with wheelchair 3. Yes with help of another person 4. Yes (with aids) |
| Activities of daily living (ADL) **CEN, SP** | - Barthel index questionnaire: score range: 0-20, interpretation according to Post et al., [[22](#_ENREF_22)]  1. 0-4: fully dependent 2. 5-9: very dependent 3. 10-14: partially independent 4. 15-19: needs minimal help with ADL 5. 20: independent   We used the 10-item Dutch Barthel Index (BI) interview version for measuring independency in performing activities of daily living (ADL), i.e. toilet use, feeding, bathing, getting dressed, and mobility [[23](#_ENREF_23), [24](#_ENREF_24), [22](#_ENREF_22)]. When the participants were care dependent, we asked a health care professional or a close family member to fill in the 10-item observation version of the BI, when possible [[25](#_ENREF_25)]. For group analyses scores for both tests were used. |
| Total hours of care per week **CEN** | - Total hours of care or assistance services needed per week:   A shower once a week (0.5 h)  Daily putting on/taking off compression stockings (1.2 h)  Daily getting up and dressing (2.3 h)  Daily going to bed (2.3 h)  Daily shaving and/or washing (1.2 h)  Toilet visits (2.3 h); transfers (2.3 h)  Daily dispense of medication (0.6 h)  The duration of complete assistance was estimated as 14 h. We ask from what age they received this assistance and whether the centenarian cooks him/herself or receives a daily meal service (see independent living). |
| Blood pressure and heartbeat **CEN, SP** | - Systolic blood pressure - Diastolic blood pressure - Heartbeat   We measure blood pressure using an OMRON M3 automatic blood pressure monitor. We ensure the participant is seated for a minimum of 5 minutes before measurements. In case systolic blood pressure is higher than 180, we measure again during the second baseline visit. When blood pressure deviates from norms we report this to the GP. |
| Grip Strength **CEN, SP** | Grip Strength is measured using a JAMAR hand dynamometer [[26](#_ENREF_26)]. We use verbal encouragement while measuring the grip-strength in the left and right hand, in duplicate. |
| Sleeping habits **CEN** | At baseline, we assess sleeping habits using the Pittsburg Sleep Quality Index questionnaire [[27](#_ENREF_27)]. Moreover we ask the centenarian since what age he/she sleeps/takes naps during the day, and how long and during which periods of the day. |

| Collected Biomaterials Sept 1^st^, 2018 | | |
| --- | --- | --- |
| Blood sample **CEN, SP, CP** | Centenarians: N=332  Partners N=2  Children: N=117  Children-partners: N=54  Siblings: 14 | Since 2013: For all subjects we collect a 65 ml blood sample used for DNA isolation, peripheral blood mononucleated cells (PBMCs) and plasma, serum, hormone levels and routine blood testing. When consent is given, a subset of PBMC cells will be used to generate iPS cells. |
| Genetic: WES **CEN** | N=276 (centenarians) | Whole exome sequencing (WES) (10Gb/exome)  Illumina HiSeq PE150, Agilent SureSelect Human All Exon V6  Illumina HiSeq PE150, NimbleGen SeqCap EZ Exome v3 (64 Mb) |
| Genetic: GWAS **CEN** | N=310 | Illumina Global Screening Array  GSAsharedCUSTOM_20018389_A2 |
| Genetic: APOE **CEN** | N=310 | Imputed from GWAS using by comparing variant ID, strand and allele frequencies to the haplotype reference panel (HRC v1.1, April 2016).  or:  Sanger sequencing of the codons 112 and 158 of the APOE gene. For this, a 428 bp fragment was generated from genomic DNA by PCR, checked for size (Fast DNA analysis with QIAxcel), and sequenced (BigDye Terminator v3.1 Cycle Sequencing kit followed by ABI 3130XL Genetic Analyzer). |
| Post-mortem brain donation (optional)  **CEN** | Total donors: N=93  Came to autopsy: N=59 | - NBB registration number of participant - NBB brain identification number - Brain weight - Post-mortem delay (time) - Neuropathology (Braak Stage, Thal stages etc.)   Since 2013: When the centenarian expresses interest in post-mortem brain donation we leave an ICF for brain donation and a Netherlands Brain Bank (NBB) information folder [[28](#_ENREF_28), [29](#_ENREF_29)]. NBB performs the brain autopsies for the 100-plus Study, and autopsies are performed according to NBB standards. Brain tissues are biobanked according to NBB protocols. In a small country such as the Netherlands (see Fig S2A), centenarians live relatively close to our center, allowing autopsy to take place usually within 8 hours after death. Neuropathologists and researchers are on call to secure brain tissues for biobanking and fresh brain tissues can be collected for experiments for which this is necessary. Brain tissues are examined based on common neuropathology hallmarks associated with neurodegenerative diseases. In March 2018, comprehensive neuropathology examination was performed for 40 centenarian brains. |
| PET-MRI or PET-CT brain imaging (optional )  **CEN** | N=9 | Collection started in February 2018 as part of the 100-plus Study phase-2; 4 centenarians were scanned before. During the baseline visit, the centenarian participants are informed about the option of PET-MRI or PET-CT brain scanning at the department of Radiology & Nuclear Medicine at our hospital. Scans will be performed to (i) detect the presence of amyloid beta accumulation in the brain and (ii) for visualizing hyperintensities, microbleeds, global atrophy, volumes of different brain structures, whole brain grey matter and cortical thickness. This visit is optional and not required for study participation. |
| Gut Microbiome (optional)  **CEN** | N=10 | Since February 2018: At baseline visits all centenarian participants are informed about the option for feces donation, for the analysis of the gut microbiome. This is optional and not required for study participation. Feces are stored in the VUmc biobank.  Questionnaire: date/time of feces, antibiotic use last half year, use of probiotics products/ visits to foreign countries, Bristol Stool Chart [[30](#_ENREF_30)]. |

# Supplementary Figures

## Fig. S1 Demographics of first 300 centenarians from 100-plus Study cohort

***A.*** *Age at study inclusion of centenarians;* ***B:*** *Birth year of centenarians included in the cohort;*

## Fig S2 Catchment Area by birth municipality.

***A.*** ***Catchment area-by-birth municipality:*** *The size of the red dots represents the number of centenarians.* ***B.*** *Inclusion rate by birth province: A per-province comparison: The number of centenarian-births was divided by the total number of births between 1910-1917, *10,000. We assumed that the chance to become a centenarian is equal across provinces. Centenarians from the provinces Limburg and Utrecht are relatively underrepresented in the cohort, while the centenarians from Drenthe are overrepresented. Birth statistics taken from Statistics Netherlands: Statistiek van den loop der bevolking van Nederland 1875-1941.*

## Fig S3 Baseline presentation of centenarians

*****Researcher subjective impression of:* ***A.*** *hearing;* ***B.*** *vision;* ***C.*** *mobility;* ***D****. independence in living circumstances;* ***E.*** *independence in* *activities of daily living* ***(****Barthel Index scores: 15-19 minimal help necessary, 20 fully independent).* ***F.*** *Geriatric depression scale (GDS) scores of centenarians (score 0: no symptoms of depression, scores >6 is suggestive of a prevalent depression).* ***G.*** *Multi-morbidities: fraction of centenarians whose GP reports mention diseases that belong to a specific number of separate disease categories.*

## Fig S4 Lifestyle and socioeconomic characteristics of centenarians

** *A.*** *The level of education of male and females was compared to the population born between 1912-1916; basic or less (primary school or less); low (1 to 2 years secondary, unfinished); medium low (3 or 4 years secondary, or lower technical school); medium (5 to 6 years secondary, or medium technical/vocational); semi-high (applied sciences or first year university); high (university or alike).* ***B.*** *Social-economic class of centenarian-fathers compared to fathers of individuals born between 1910-1915; and social economic class of centenarians as adults compared to males born between 1910-1919.* ***C.*** *Number of children parented by centenarians, compared to individuals born between 1910-1915.*

## Fig S5 APOE allele distribution of centenarians in 100-plus Study cohort

*Number of children parented by centenarians, compared to individuals born between 1910-1915.*

# References

1. Last JM. Dictionary of Epidemiology. 4th ed. New York: Oxford University Press; 2001.

2. Fay MP, Brittain EH, Proschan MA. Pointwise confidence intervals for a survival distribution with small samples or heavy censoring. Biostatistics. 2013;14(4):723-36.

doi:10.1093/biostatistics/kxt016.

3. Central Bureau of Statistics: Life expectancy per birth cohort. [database on the Internet]. Netherlands Statistics. 2017. Available from:

http://statline.cbs.nl/Statweb/dome/?TH=26190&PA=80333NED&LA=nl. Accessed: 11 Aug 2015

4. Fergany N. On the human survivorship function and life table construction. Demography. 1971;8(3):331-4.

5. Gompertz B. On the Nature of the Function Expressive of the Law of Human Mortality, and on a New Mode of Determining the Value of Life Contingencies. Philosophical Transactions of the Royal Society of London. 1825;115. :513–83.

6. Gavrilov LA, Gavrilova NS. Mortality Measurement at Advanced Ages: A Study of the Social Security Administration Death Master File. North American actuarial journal : NAAJ. 2011;15(3):432-47.

7. Burns RA, Butterworth P, Kiely KM, Bielak AA, Luszcz MA, Mitchell P et al. Multiple imputation was an efficient method for harmonizing the Mini-Mental State Examination with missing item-level data. Journal of clinical epidemiology. 2011;64(7):787-93. doi:10.1016/j.jclinepi.2010.10.011.

8. Verhage F. Doctoral dissertation: Intelligence and age: Research with Dutch people aged 12 to 77. Assen: Van Gorcum.: University of Groningen; 1964.

9. UNESCO. International Standard Classification of Education 1997.

http://www.unesco.org/education/information/nfsunesco/doc/isced_1997.htm. 1997.

10. Vliegen JM, de Jong U, Wesselingh AA, van der Kley P, CBS, SISWO. Education in the Netherlands. Monografiëën volkstelling 1971. The Hague s-Gravenhage: Centraal Bureau voor de Statistiek: Staatsuitgeverij; 1981.

11. HSN standardized, HISCO-coded and classified occupational titles, release 2013.01 [database on the Internet]. IISG. 2013. Accessed:

12. van Leeuwen MHD, Maas I, Miles A. HISCO. Historical International Standard Classification of Occupations. 2002.

13. Van Leeuwen MHD, Maas I. HISCLASS. A Historical International Social Class Scheme. Leuven: University Press Leuven; 2011.

14. Folstein MF, Folstein SE, McHugh PR. "Mini-mental state". A practical method for grading the cognitive state of patients for the clinician. Journal of psychiatric research. 1975;12(3):189-98.

15. Kok RM, Verhey FRJ. Gestandaardiseerde MMSE. Altrecht GGZ. 2002.

16. Tombaugh TN, McIntyre NJ. The mini-mental state examination: a comprehensive review. Journal of the American Geriatrics Society. 1992;40(9):922-35.

17. de Jonghe JF, Schmand B, Ooms ME, Ribbe MW. [Abbreviated form of the Informant Questionnaire on cognitive decline in the elderly]. Tijdschrift voor gerontologie en geriatrie. 1997;28(5):224-9.

18. Jorm AF, Jacomb PA. The Informant Questionnaire on Cognitive Decline in the Elderly (IQCODE): socio-demographic correlates, reliability, validity and some norms. Psychological medicine. 1989;19(4):1015-22.

19. de Jager CA, Budge MM, Clarke R. Utility of TICS-M for the assessment of cognitive function in older adults. International journal of geriatric psychiatry. 2003;18(4):318-24. doi:10.1002/gps.830.

20. Yesavage JA, Sheikh JI. 9/Geriatric Depression Scale (GDS). Clinical Gerontologist. 1986;5(1-2):165-73. doi:10.1300/J018v05n01_09.

21. Bleeker JAC, de Winter FML, Cornelissen E. Geriatric Depression Scale. 1985.

22. Post MW, van Asbeck FW, van Dijk AJ, Schrijvers AJ. [Dutch interview version of the Barthel Index evaluated in patients with spinal cord injuries]. Nederlands tijdschrift voor geneeskunde. 1995;139(27):1376-80.

23. Mahoney FI, Barthel DW. Functional Evaluation: The Barthel Index. Maryland state medical journal. 1965;14:61-5.

24. Collin C, Wade DT, Davies S, Horne V. The Barthel ADL Index: a reliability study. International disability studies. 1988;10(2):61-3.

25. de Haan R, Limburg M, Schuling J, Broeshart J, Jonkers L, van Zuylen P. [Clinimetric evaluation of the Barthel Index, a measure of limitations in dailly activities]. Nederlands tijdschrift voor geneeskunde. 1993;137(18):917-21.

26. Bechtol CO. Grip test; the use of a dynamometer with adjustable handle spacings. The Journal of bone and joint surgery American volume. 1954;36-A(4):820-4; passim.

27. Buysse DJ, Reynolds CF, 3rd, Monk TH, Berman SR, Kupfer DJ. The Pittsburgh Sleep Quality Index: a new instrument for psychiatric practice and research. Psychiatry research. 1989;28(2):193-213.

28. Netherlands Brain Bank. http://www.brainbank.nl/.

29. Ravid R, Swaab DF. The Netherlands brain bank--a clinico-pathological link in aging and dementia research. Journal of neural transmission Supplementum. 1993;39:143-53.

30. Lewis SJ, Heaton KW. Stool form scale as a useful guide to intestinal transit time. Scandinavian journal of gastroenterology. 1997;32(9):920-4. doi:10.3109/00365529709011203.
